# Blastomycosis in Ontario, 1994–2003

**DOI:** 10.3201/eid1202.050849

**Published:** 2006-02

**Authors:** Shaun K. Morris, Jason Brophy, Susan E. Richardson, Richard Summerbell, Patricia C. Parkin, Frances Jamieson, Bill Limerick, Lyle Wiebe, E. Lee Ford-Jones

**Affiliations:** * Hospital for Sick Children, Toronto, Ontario, Canada;; † Ontario Ministry of Health and Long-term Care, Toronto, Ontario, Canada;; ‡ Northwestern Ontario Health Unit, Kenora, Ontario, Canada

**Keywords:** Blastomycosis, Ontario, Pediatrics

## Abstract

We describe a case of blastomycosis in an 8-year-old boy with *Blastomyces*-associated osteomyelitis and possible pulmonary involvement. We also identify 309 cases of blastomycosis in Ontario that were seen during a 10-year period, 57% of which occurred from 2001 to 2003. The overall incidence during the study period was 0.30 cases per 100,000 population. Most patients were from north Ontario (n = 188), where the incidence was 2.44 cases per 100,000. The incidence in the Toronto region was 0.29 per 100,000. Thirteen percent of cases occurred in children <19 years of age. These findings substantially increase the number of known cases in Ontario and Canada. Clinicians may encounter persons infected with *Blastomyces dermatitidis* and must be familiar with its signs and symptoms and be aware of locations, such as northwestern Ontario, where disease is endemic or hyperendemic. We advocate resuming blastomycosis as a reportable disease in Ontario to facilitate tracking cases.

## Abstract 2

Clinicians in Ontario should be aware of symptoms and areas where disease is endemic.

First described by Gilchrist in 1894 (*1*), blastomycosis has been documented in Canada since at least 1910 (*2*). The incidence and epidemiologic features of the disease are poorly understood because of underrecognition, difficulty in isolating *Blastomyces dermatitidis* from natural sites, lack of an effective skin test, and because blastomycosis is not nationally reportable in either Canada or the United States (*3*). Blastomycotic infections in Canada have recently been reported in international (*4*) and Canadian (*5**–**7*) literature. We report a case of pediatric blastomycotic osteomyelitis and the results of an almost 10-year review of *Blastomyces* infection in Ontario through reports of laboratory isolates submitted to the Central Public Health Laboratory (CPHL), Ontario Ministry of Health and Long-term Care, Toronto. The objectives of the review were to define in the province of Ontario 1) the geographic epidemiologic features of laboratory-confirmed blastomycosis, 2) changes in the number of cases over time, and 3) demographic characteristics of infected persons. The case report and study were approved by the research ethics board of the Hospital for Sick Children, Toronto, Ontario.

## Case Report

An 8-year-old, previously healthy Caucasian boy was brought to his family physician with a 2-month history of neck pain and stiffness. The pain was not sufficient to wake the child at night, but it prevented participation in athletics. No history of trauma, fever, weakness, paresthesias, weight loss, or change in bowel or bladder function was noted. The initial diagnosis was muscular strain, and rest and antiinflammatory medication was recommended. When his symptoms did not improve, a cervical spine radiograph showed a lytic lesion of the fifth cervical vertebra.

On hospital admission, physical examination was unremarkable, with the exception of pain on palpation over the posterior cervical spine. Laboratory results at admission showed normal leukocyte count (8.0 × 10^9^/L), differential (polymorphs 4.48 × 10^9^/L, eosinophils 0.08 × 10^9^/L, lymphocytes 2.80 × 10^9^/L, monocytes 0.56 × 10^9^/L), electrolytes, and renal function. Erythrocyte sedimentation rate was mildly elevated at 38 mm/h. Computed tomographic scan of the region showed a well-defined lytic lesion with a "bubbly" appearance involving the posterior elements of the C5 vertebral body. An incidental note was made of a small, nonspecific lesion within the posterior upper lobe of the right lung. Bone scintillography showed positive uptake at C5. Magnetic resonance imaging (MRI) of the cervical spine demonstrated an enhancing mass that involved the posterior aspects of C5 plus an abnormal signal within the adjacent spinous processes.

The patient underwent a C5 laminectomy and a C4 partial hemilaminectomy. During dissection, a small amount of purulent liquid was extruded from the tissue above C5. Gram stain of the pus aspirate showed no organisms. A calcofluor stain was positive for large, broad-based budding yeast forms. Pathologic examination showed a destructive lesion involving bone and soft tissues with both granulomatous and necrotizing suppurative components. The numerous epithelioid granulomas contained Langhans cells and foreign body giant cells, while the necrotizing lesions contained neutrophilic and eosinophilic infiltrates. Fungal elements highlighted with periodic acid–Schiff and Gomori methenamine silver stains showed double-contoured cell walls surrounding a cytoplasmic mass and proliferation in the form of broad-based buds. On the basis of these features, a presumptive diagnosis of blastomycosis was made. Subsequent fungal cultures grew a filamentous dimorphic fungus identified as *B. dermatitidis* and confirmed by DNA probe (Accuprobe, Gen-Probe Inc., San Diego, CA, USA).

The lung lesion in the right upper lobe could have also been a focus of blastomycotic infection; however, bronchoalveolar lavage or biopsies were not performed. Intravenous amphotericin B, 30 mg every 24 h (1.0 mg/kg/d) was initiated. After 10 days, the dose was reduced by 50% because of renal toxicity. On day 17 of treatment, itraconazole (200 mg orally once per day) was initiated and given concurrently with amphotericin B for 5 days, at which point amphotericin B was discontinued because of laboratory evidence of renal failure. At the time of discontinuation, 480 mg (16 mg/kg) had been given. The patient's clinical status improved, with resolution of pain and a return of full cervical range of motion by time of discharge.

Three weeks after completing a 6-month course of itraconazole, fever associated with vomiting and a sore throat developed in the patient. Chest radiograph showed a consolidation in the upper lobe of the right lung. MRI of the lung fields and cervical spine did not provide evidence of recurrence of blastomycosis. Clarithromycin was initiated and continued for 3 days with no improvement, at which time itraconazole (200 mg orally once per day) was resumed because blastomycosis could not be conclusively ruled out. Antifungal therapy was continued for 6 months, after which the patient was clinically healthy, and radiographs of the cervical spine and chest were normal.

## Field Epidemiologic Investigation of Case

The patient had visited a cottage on the lakeshore of an island in the north health region of Ontario 5 months before his hospitalization. Shortly after the owner purchased the property, his dog and an adult male companion both developed laboratory-confirmed, nonfatal blastomycosis. Six months later, another adult male visitor developed a pneumonialike condition and was diagnosed with laboratory-confirmed blastomycosis. A provincial epidemiologic team also determined that a dog belonging to a previous owner of the property died of blastomycosis ≈2 years earlier.

A year and a half after the initial human infection, the cottage owner contacted the mycology laboratory at the Ontario Ministry of Health and requested an environmental investigation. A total of 50 environmental samples were taken from the property, including from a beaver lodge (similar to those previously associated with blastomycosis [8]) located underneath a boathouse. The samples were taken ≈3 months after our patient's presumed exposure. Material from the samples was prepared in sterile physiologic saline and injected into 4 mice per sample, as outlined by Ajello and Weeks (*9*) for environmental isolation of *Histoplasma capsulatum*. This technique had previously been used successfully to isolate *B. dermatitidis* (*10*). No mice died of blastomycosis within 6 weeks (autopsy of 2 mice that died showed bacterial infection), and examination of the remaining 198 mice, euthanized after 6 weeks, showed that livers, spleens, and lungs were clear of *B. dermatitidis* by histopathology and by culture on Sabouraud glucose agar with cycloheximide, chloramphenicol, and gentamicin (CCG) and blood agar with CCG and 2.3% egg albumin (*11**,**12*).

## Laboratory-based Review of Blastomycosis in Ontario, 1994–2003

### Methods

Cases were defined as all positive cultures of *B. dermatitidis* isolated between November 1, 1994, and December 31, 2003. CPHL processes primary cultures cultivated from patient samples sent from referring facilities. It also confirms the identity of cultures sent from referring laboratories. Eleven public health laboratories are in Ontario, and all of these laboratories, except the Thunder Bay laboratory, refer all isolates of *B. dermatitidis* to the CPHL for confirmation. Contact was made with the Northwestern Ontario Health Unit in Kenora, Ontario, to obtain records of confirmed cases of blastomycosis that may not have been sent to CPHL. Some of these cases (and their isolates) are referred to Winnipeg rather than southern Ontario, and some isolates are identified only at the Thunder Bay laboratory. By contacting the specific region that does not refer all cases to the CPHL, a high level of case capturing is ensured. Information obtained regarding each infected person included date of birth, sex, and location of diagnosis. Ontario is divided into 7 health regions (Toronto, southwest, central-south, central-west, central-east, east, and north). Incidence per 100,000 population per year was derived by using population data from Statistics Canada (B. Ball, pers. comm.).

## Results

**Figure 1:**
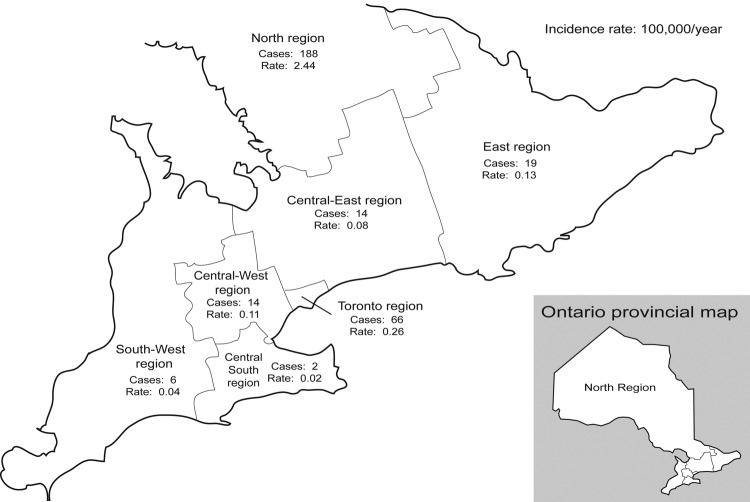
Incidence of blastomycosis by Ontario health region.

A total of 309 culture-positive cases of blastomycosis were identified in Ontario during the study period. Each of Ontario's 7 health regions had cases identified (Figure 1); 61% (n = 188) of the cases were diagnosed in the north region, and 21% (n = 66) were diagnosed in the Toronto region. Of the north region cases, 89% (n = 167) were from the Northwestern Health Unit's district, which includes Kenora.

**Figure 2:**
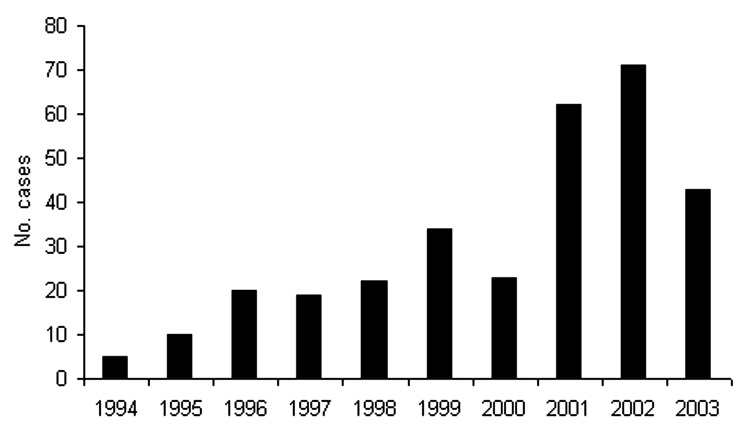
Blastomycosis diagnosed by year, Ontario, 1994–2003.

**Figure 3:**
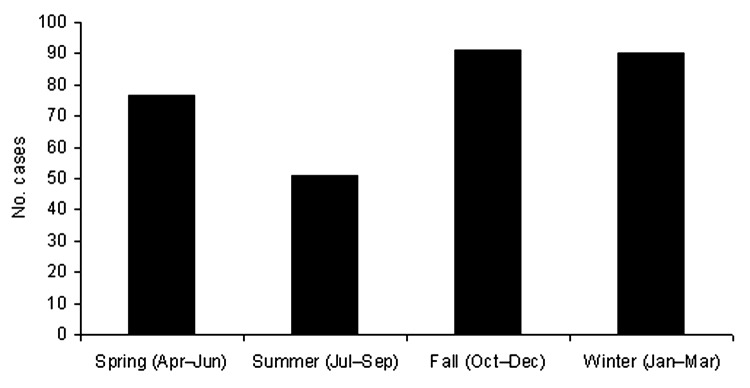
Blastomycosis diagnosed by season, Ontario, 1994–2003.

**Figure 4:**
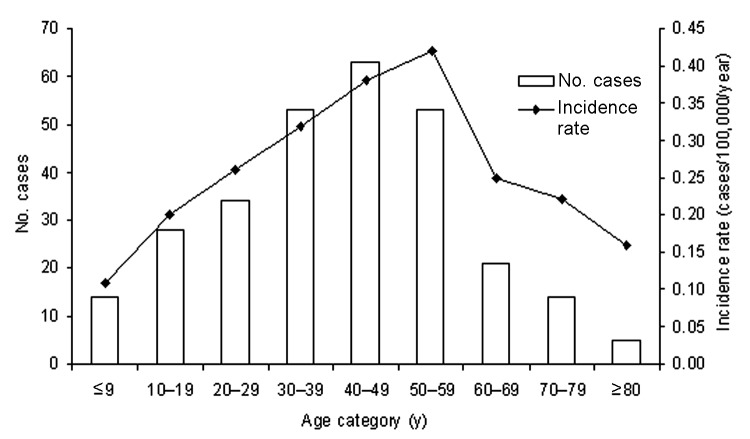
Number of blastomycosis cases and incidence rates by age, Ontario, 1994–2003.

The mean number of cases diagnosed per year was 33.7; the fewest number of cases was in 1995 (n = 10), and the greatest number was in 2002 (n = 71) (Figure 2). The number of cases has increased in recent years; 57% (n = 175) were reported in the 3-year period from 2001 to 2003. While cases were diagnosed year-round, 59% (n = 181) occurred in the fall and winter months, from October to March (Figure 3). The age of patients with positive isolates was recorded for 92% (n = 283) of cases; ≈60% were 30–59 years of age (range 6 months to 83 years) (Figure 4). The sex of the affected person was available in 97% (n = 301); 65% (n = 196) were male, and 35% (n = 105) were female. The incidence ranged from a low in the south-central Ontario region of 0.02 cases per year per 100,000 population to a high in the north Ontario region of 2.44 cases per year per 100,000 population (Figure 1). Age-specific incidence rates are shown in Figure 4.

## Discussion

### Case Report

Blastomycosis is a rare but potentially fatal infection caused by the thermally dimorphic fungus *B. dermatitidis*. It is presumed to be a soil organism, and factors that favor it include moisture, high content of organic material, acidic pH, and exposure to animal excreta (*8**,**13*). Primary infection generally follows inhalation of conidia, asexual fungal spores that are shed at maturity. Blastomycosis often has nonspecific initial symptoms: fever, malaise, myalgias, weight loss, cough, and pleuritic chest pain (*14*). The course of illness may be either acute or chronic. While any organ may be involved, lung involvement is most common, manifesting either as a lobar infiltrate resembling a bacterial pneumonia, a miliary infiltration similar to tuberculosis, or as a large mass that is initially suspected of being a bronchogenic carcinoma. The second most common type of disease is cutaneous. Less common clinical syndromes involve bone, the genitourinary system, or the central nervous system (CNS). In contrast to most other invasive fungal infections, blastomycosis most commonly affects immunocompetent persons.

Children make up 2%–11% of cases in previous studies (*15**,**16*). Published case reports and case series of pediatric blastomycosis highlight the potential for pulmonary, bone, and CNS disease, as well as neonatal disease and intrauterine transmission (*6**,**15**–**20*). In our review, 13% of patients were <19 years of age.

Recent guidelines from the National Institute of Allergy and Infectious Diseases Mycoses Study Group and the Infectious Diseases Society of America suggest that life-threatening pulmonary or disseminated non-CNS disease, any CNS disease, and disease in the immunocompromised host be treated with amphotericin B to complete a total dose of 1.5–2.5 g (21–36 mg/kg for a 70-kg person). For patients who do not tolerate amphotericin, it can be used initially until the condition has stabilized at which point itraconazole (for non-CNS disease) or high-dose fluconazole (for CNS disease) can be used as stepdown therapy (*21*). Mild-to-moderate pulmonary and disseminated non-CNS disease can be treated by itraconazole from onset. Duration of therapy with itraconazole should be >6 months, except in the case of osteomyelitis, which has a higher rate of relapse (*22*). Bone disease should be treated with >1 year of antifungal medications. Relapse is a recognized outcome in blastomycosis and may occur after any treatment regimen (*22**–**24*). Therefore, in our case, given the vertebral involvement, 1 year of itraconazole therapy was warranted.

### Epidemiologic Report

*B. dermatitidis* is endemic in the United States around the Mississippi and Ohio River basins, Midwestern states, Canadian provinces bordering the Great Lakes, and small areas bordering the St. Lawrence River (*25*). Ontario has an area >1 million km^2^ and can be divided into 3 natural regions: the rolling uplands of the Canadian shield across the center of the province, the Hudson Bay lowlands to the north, and the Great Lakes and St. Lawrence lowlands to the south. The northwestern portion of the province is largely forested and dotted with lakes and rivers.

While the epidemiologic investigation described in our case failed to yield positive environmental cultures, the link between beaver dwellings and blastomycosis has previously been suggested (*8*). Other outbreaks have been reported in association with construction of a log cabin (*26*), an urban construction site (*27*), outdoor riverside camping (*28*), raccoon hunting (*29*), and travel to a specific small island (*30*).

Blastomycosis was a reportable disease in Ontario until 1989 (*31*). Most Canadian cases have been reported from Ontario and Manitoba (*3**,**11**,**31**–**35*). From 1981 to 1989, before the removal of blastomycosis from the list of reportable diseases, 16 cases were recorded in Ontario, a mean of 1.8 cases per year (*31*). The current review suggests that blastomycosis is more common than previously thought, with a mean of 33.7 cases diagnosed per year in the 10-year study period. Hyperendemicity in the region surrounding Kenora, Ontario, has recently been reported (*35*), with an estimated annual incidence rate of 117.2 cases per 100,000 population (*35*). This amount exceeds the next highest rate reported in North America of 100 cases per 100,000 population in the Eagle River area in Vilas County, Wisconsin (*36*). An increase was noted in the number of cases of blastomycosis identified per year since the late 1990s (Figure 2). Increased awareness in Kenora because of an education campaign aimed at physicians after a fatal case of blastomycosis in 1998 may account in part for the increased number of recognized cases. Our identification of 309 laboratory-confirmed cases of blastomycosis represents the largest group of confirmed cases and substantially increases the total number of known cases in both Ontario and Canada.

During the study period, 66 cases were diagnosed in Toronto. While blastomycosis has previously been reported in persons who have not traveled outside of the greater Toronto area (*5**,**6*), we could not confirm where infection was acquired in the 66 Toronto patients. The major limitation of this study is that the geographic data are based on location of diagnosis, which may not be reflective of location of infection. Infection may occur in rural regions that are frequent destinations of travel for many Ontario residents.

The preponderance (58%, p = 0.0005) of diagnoses in fall and winter noted in our study has previously been reported (*35*). The incubation period for symptomatic blastomycosis is 1–3 months (*13**,**37*). Therefore, infection is thought to mainly occur in summer and fall, when persons spend time outdoors, snow cover is minimal, and rainfall is high, thereby increasing exposure to this soil organism (*8**,**13*).

Early Canadian studies (*2**,**33*) found most (85%–88%) cases to be in males, and early studies from the United States suggested a male-to-female ratio of 4:1–15:1 (*38*). More recent studies in Canada (*31**,**35*) and the United States (*39*) suggest that the male sex predominance is not as large. In our study, 65.1% of patients were male, similar to the proportion noted by Crampton et al. (*32*).

Previous studies have suggested increased rates of infection for both African Americans (*40*) and Aboriginals (*35*). Whether this finding is related to genetic or exposure factors is unclear. Given the retrospective nature of this laboratory review, we could not determine the ethnicity of the cases. Future work must identify high-risk groups within the population so that targeted prevention efforts may be put in place.

Despite being the largest series of cases of blastomycosis reported in Ontario, our data may underestimate the true incidence of disease in the province. The number of cases diagnosed in northwestern Ontario increased after an education campaign. To our knowledge, no similar campaign has been carried out elsewhere in the province, and therefore the diagnosis is likely often missed. Some samples may have been only identified at a regional laboratory and not counted among our data. Because of the geographic proximity, patients in northwestern Ontario are often transferred to Winnipeg, Manitoba, rather than to an Ontario tertiary centre for investigation and treatment. Additionally, persons from outside the province who are infected may be diagnosed in their home provinces. As a result, some cases of blastomycosis acquired in Ontario may be diagnosed in Manitoba or elsewhere and therefore are not included in our data.

## Conclusion

The understanding of the natural distribution of blastomycosis and other mycoses endemic in Ontario (such as histoplasmosis) is minimal. This study is the first to describe the Ontario-wide incidence of blastomycosis and to provide incidence rates in each of the 7 provincial health regions. Clinicians practicing throughout the province and country may encounter persons infected with this organism and need to be familiar with its varied clinical signs and symptoms and be aware of regions where disease is endemic or hyperendemic. Our data suggest that the number of diagnoses of blastomycosis has increased over several years. However, the disease likely remains underrecognized. As delay to diagnosis can contribute to illness and death, clinicians should consider blastomycosis in their differential diagnoses of lung, skin, and bone diseases, particularly if the patient does not respond to conventional antimicrobial drug therapy. The lack of rapid and effective diagnostic tools contributes to the underrecognition of blastomycosis. Advances in molecular diagnosis of *B. dermatitidis* (*39*), particularly in regions identified as higher risk, hold the potential for improving case detection and decreasing delay to diagnosis.

Infection by *B. dermatitidis* is more common than was thought before its removal from the list of reportable diseases in Ontario in 1989. Our group advocates strongly for returning blastomycosis to the reportable diseases list in this province. Travel history must be included in the reporting of blastomycosis. While identifying the point of infection in well-traveled individuals may be impossible, a specific or negative travel history would make a valuable contribution to understanding where blastomycosis is contracted in Ontario. Such reporting would facilitate tracking cases and clinical education regarding this potentially fatal invasive fungal infection.
